# Early changes in scores of chronic damage on transplant kidney protocol biopsies reflect donor characteristics, but not future graft function

**DOI:** 10.1111/ctr.12251

**Published:** 2013-10-09

**Authors:** Ben Caplin, Kristin Veighey, Arundathi Mahenderan, Miriam Manook, Joanne Henry, Dorothea Nitsch, Mark Harber, Peter Dupont, David C Wheeler, Gareth Jones, Bimbi Fernando, Alexander J Howie, Peter Veitch

**Affiliations:** a Centre for Nephrology, UCL Medical SchoolLondon, UK; b Renal and Transplant Unit, Royal Free London NHS Foundation TrustLondon, UK; c Department of Non-Communicable Disease Epidemiology, Faculty of Epidemiology and Population Health, London School of Hygiene and Tropical MedicineLondon, UK; d Department of Pathology, University College LondonLondon, UK

**Keywords:** chronic allograft injury, chronic damage index, digital image analysis, protocol biopsy, renal transplantation

## Abstract

The amount of irreversible injury on renal allograft biopsy predicts function, but little is known about the early evolution of this damage. In a single-center cohort, we examined the relationship between donor-, recipient-, and transplantation-associated factors and change in a morphometric index of chronic damage (ICD) between protocol biopsies performed at implantation and at 2–3 months. We then investigated whether early delta ICD predicted subsequent biochemical outcomes. We found little evidence to support differences between the study group, who had undergone serial biopsies, and a contemporaneous control group, who had not. In allografts with serial biopsies (n = 162), there was an increase in ICD between implantation (median: 2%, IQR:0–8) and 2–3 months post-transplant (median 8% IQR:4–15; p < 0.0001). Donation from younger or live donors was independently associated with smaller early post-transplant increases in ICD. There was no evidence for a difference in delta ICD between donation after cardiac death vs. donation after brain death, nor association with length of cold ischemia. After adjustment for GFR at the time of the second biopsy, delta ICD after three months did not predict allograft function at one yr. These findings suggest that graft damage develops shortly after transplantation and reflects donor factors, but does not predict future biochemical outcomes.

Improvements in the outcomes of kidney transplantation have mainly been confined to prevention of early graft loss. Over the last 20 yr, the slopes of allograft survival curves from one yr post-transplant have remained almost parallel 1,2. Furthermore, due to the chronic undersupply of donor organs, there has been a recent move toward the use of “extended criteria” donors and donation after cardiac death (DCD) with consequent concerns surrounding the long-term outcomes for recipients of these kidneys.

The total amount of irreversible histological damage on protocol biopsies at early time points, as estimated by Banff criteria for chronic damage 3–5 or as measured morphometrically 6,7, is a predictor of longer-term graft outcomes, and this damage appears to be established early post-transplantation.

Although previous studies have examined clinical predictors of chronic damage at a single time point or over many years, little is understood about the factors surrounding transplantation that are associated with the early evolution of such damage, that is, the relationship between clinical variables and changes in measures of chronic damage during the first post-transplant months. This is an issue of particular importance in kidneys from “extended criteria” donors (ECD) or donation following cardiac death (DCD) as these allografts are likely to have significant levels of pre-existing chronic damage in the former case and suffer increased warm ischemic injury in the latter. The use of a quantitative approach to assess damage, made possible by the use of digital analysis systems, has been explored in an investigation of recipients of live donor transplants 8 or kidneys donated following brain death (DBD) 9, but the risk factors for the early evolution of chronic damage on protocol biopsy of single-organ kidney transplants from a cohort receiving kidneys of differing donor types remain unclear.

Using protocol biopsies performed as a part of routine clinical care, the aim of this study was to examine donor and recipient factors associated with changes in a morphometric measure of chronic damage over the first three post-transplant months. We then investigated whether the early change in this same measure was a useful predictor of graft function at one yr.

## Patients and Methods

In this cohort study, adult recipients of solitary renal allografts under the care of the Royal Free London NHS Foundation Trust (RFLNHSFT), transplanted between February 1, 2008, and October 31, 2010, underwent protocol biopsies as part of their routine clinical care. The unit policy is for protocol biopsies to be performed at three time points: 30 min following allograft implantation (PB0), 6–12 wk post-transplantation (PB1), and one yr post-transplantation. Only data from the first two are described here, as uptake of the one-yr biopsy is low (n = 72, 29%) and likely to be heavily influenced by clinical factors; that is, those patients with a well-functioning graft are likely to forego this biopsy. All patients undergoing biopsy gave informed consent as to the clinical risks and benefits of the program. The following were considered contraindication to protocol biopsy: high bleeding risk, pregnancy, and indicated biopsy within the last 14 d.

The immunosuppressive protocol was unchanged for the duration of this study. Induction therapy consisted of basiliximab (days 0 and 4), methylprednisolone, tacrolimus, and mycophenolate mofetil. Maintenance therapy (commenced five d prior to the transplant in recipients of live donor kidneys) consisted of (i) tacrolimus adjusted to maintain a trough level of 9–12 μg/L for the first three months and then 6–9 μg/L (except in cases of delayed graft function where target trough levels are 6–9 μg/L until graft function is established); (ii) mycophenolate mofetil 1 g bd reduced to 750 mg bd after one month; (iii) prednisolone 20 mg daily for one wk and 10 mg daily for one wk followed by steroid withdrawal. Acute cellular rejection was treated with pulsed methylprednisolone and continuation of oral steroids with the addition of antithymocyte globulin (ATG) in resistant cases. Acute vascular rejection was treated with the addition of plasma exchange where there was an identifiable donor-specific antibody, plus rituximab in resistant cases. Patients underwent a preemptive CMV surveillance strategy (except for those enrolled in a clinical trial or considered high risk), receiving antiviral therapy only where routine samples revealed CMV antigen levels >1250 copies/mL.

Protocol biopsies were performed by senior clinicians, under direct vision at implantation or live ultrasound guidance post-transplantation. Samples were obtained using either 16-gauge needles (at implantation) or 18-gauge biopsy needles (post-transplant) and were examined using a dissecting microscope at the bedside to confirm adequate sampling. Biopsies were formalin-fixed, paraffin-embedded, and serially sectioned before staining using conventional techniques. The morphometric measure, called the index of chronic damage (ICD), was determined in sections from PB0 to PB1 (ICD0 and ICD1, respectively) as previously described 7. Briefly, on a computerized image, the area of renal cortex excluding renal capsule was measured in arbitrary units. Areas of chronic damage, identified by global sclerosis of glomeruli, tubular atrophy, interstitial fibrosis, and/or occluded vessels, were also quantified. The amount of damage was expressed as a percentage of cortical area to the nearest integer.

Donor data were retrieved from NHS Blood and Transplant (NHSBT). NHSBT definitions were used for mismatch level 10. Donor serum creatinine concentration from admission was used where available; otherwise, first available creatinine was obtained. Recipient data were retrieved from electronic hospital records. Rejection was defined as per Banff criteria on indication biopsies prior to PB1 or on that protocol biopsy (or on biopsies at less than three months for the group not undergoing PB1). BK nephropathy was defined by the presence of virus detected by an immunoperoxidase method. The number of indicated biopsies was the total number of biopsy attempts prior to the first post-transplant biopsy (or in the first three months for those not undergoing PB1). Serum creatinine values were obtained from day 56 and day 365 (or the nearest available measurement subsequent to these dates) post-transplant eGFR calculated by the 4-variable MDRD equation 11. All analyses were performed by allograft, so two patients who both received a second transplant during the study period were represented twice although sensitivity analysis excluding these second grafts did not alter any of the main findings.

As not all patients transplanted in the study period had undergone serial protocol biopsies, we examined whether there were differences between the group who had undergone such biopsies and the group who had not using the chi-squared test (Fisher’s exact test for cells with small numbers) and *t*-test (Wilcoxon rank-sum test for nonparametric variables). Then, in a sensitivity analysis, we examined whether inclusion in the biopsy group was associated with differences in eGFR at eight wks or cumulative incidence of rejection over the follow-up period, after matching using a propensity score based on donor and recipient characteristics.

We went on to examine the associations between ICD and clinical variables. ICD scores were positively skewed at all time points so to examine predictors of ICD0, and change in ICD (ICD1-ICD0; delta ICD) scores was divided into equal frequency tertiles and quartiles, respectively. Univariable associations between clinical variables and ICD0 or delta ICD were examined using analysis of variance (ANOVA) (Kruskal–Wallis [K-W] test for nonparametric variables) and chi-squared test (Fisher’s exact test for cells with small numbers). The change in ICD between paired samples was examined using the Wilcoxon rank-sum test.

For multivariate analysis, we did not group ECD and DBD donors separately, firstly to avoid small cell numbers and also so we could examine clinical factors that would lead to the definition of a donor as ECD (donor serum creatinine >133 μMol/L; intracerebral hemorrhage, ICH; donor age) in both DBD and DCD groups. To identify independent predictors of ICD0 and delta ICD, we used a logistic regression models and included exposure variables where we hypothesized an impact on ICD. For baseline chronic damage, these were donor type (live, DCD, or donation after brain death [DBD]), age, sex, ethnic group, donor serum creatinine>133 μMol/L, ICH, diabetes mellitus (DM), and cold ischemic time (CIT; deceased donors only). For change in chronic damage, these were as above plus recipient factors: mismatch level, age, sex, ethnic group, previous transplantation, primary disease, DM, BK nephropathy, and rejection. Adjustment was made for the timing of PB1, but otherwise variables were retained in the models only where there was an improvement in model fit as demonstrated by a reduction in the −2 log-likelihood ratio. We also performed a restricted analysis excluding donors dying from ICH. Following fitting of the ordinal logistic model, the proportional odds assumption was tested for each of the variables in the model individually. Where this was violated, as in the model of ICD0, a multinomial logistic approach was used, and relative risk ratios were reported for each category. Predicted odds ratios or relative risk ratios were compared with observed values for both multivariable models. A further sensitivity analysis examining change in chronic damage was carried out using a linear regression model with log ICD1 adjusted for log ICD0, and although model fit was poor, the main conclusions, as identified using the logistical modeling approach, were unchanged.

To investigate whether delta ICD might predict future outcome, we then examined whether this same measurement (as an explanatory variable) was associated with biochemical measures of kidney function. First, we examined the relationship between delta ICD quartile and quartile of GFR at biopsy. Then, to test the usefulness of the measure as a predictor of future function, we examined the association between delta ICD quartile and the change in eGFR between the time of PB1 and one-yr post-transplant (eGFR at day 365 after adjustment for eGFR at day 56).

Summary statistics are presented as means ± standard deviation (or medians with interquartile range for non-normally distributed variables). Statistical significance was accepted as p < 0.05. This was a descriptive study, and therefore, no power calculations were performed. Data were analyzed in Stata 11 (Stata Corp LP, College Station, TX, USA).

## Results

### Cohort

Two hundred and sixty seven patients under follow-up at the renal transplant unit RFLNHSFT underwent 269 kidney-alone transplants between February 1, 2008, and October 31, 2010. Six patients were transplanted elsewhere, so no ICD0 was available. A further 17 allografts were excluded from further analysis due to nephrectomy (n = 8), loss to follow-up (n = 2), recipient death (n = 5), and contraindication to protocol biopsy (n = 2). Either ICD0 (n = 45) or ICD1 (n = 61) or both were not available on a further 81 allografts due to patient refusal (n = 5), multiple recent indicated biopsies (n = 2), or undocumented reasons. Delta ICD was available for 162 allografts, and along with three allografts with biopsies that were technically inadequate, this group (n = 165) was compared with those who did not undergo both protocol biopsies (n = 81). The “6–12 wks” post-transplant protocol biopsies were performed a median of 56 d (IQR 48–72) following transplantation. There was a higher prevalence of female donors, and there were an increased number of indicated biopsies in the group who did not undergo serial biopsies, but there were no other differences observed between the groups (Table 1). Specifically, there were no differences seen in CIT, the prevalence of different donor types, eGFR, or biopsy proven rejection. In a sensitivity analysis, using propensity score matching, no significant differences were seen in eGFR at eight wk or the cumulative incidence of rejection between those undergoing protocol biopsies and those who did not (data not shown).

**Table 1:** Description of the study cohort and comparison between the groups of allografts for which serial biopsies were not and were performed

	All allografts n = 246		Allografts without serial biopsies n = 81		Allografts with serial biopsies n = 165		p-Value
Recipient age							
Years, mean, SD	49.4	13.8	47.5	15.3	50.2	13.1	NS
Recipient sex							
Female n,%	114	46.3	38	46.9	76	46.1	NS
Recipient ethnic group							
Asian n,%	51	20.7	19	23.5	32	19.4	NS
Black n,%	54	22.0	13	16.1	41	24.9	
Chin/oth/ns n,%	34	13.8	9	11.1	25	15.2	
White n,%	107	43.5	40	49.4	67	40.6	
Recipient primary disease							
Cystic n,%	20	8.1	8	9.9	12	7.3	NS
DM n,%	27	11.0	11	13.6	16	9.7	
Dysplastic/reflux n,%	10	4.1	4	4.9	6	3.6	
Glomerular n,%	48	19.5	17	21.0	31	18.8	
Vasc/isch/hyp n,%	27	11.0	9	11.1	18	10.9	
Other n,%	74	30.1	23	28.4	51	30.9	
Unknown n,%	40	16.3	9	11.1	31	18.8	
Recipient DM							
n,%	35	14.2	10	12.4	25	15.2	NS
Previous transplant							
n,%	25	11.0	12	14.8	15	9.1	NS
Donor age							
Years, mean, SD	48.0	15.8	47.3	16.3	48.3	15.5	NS
Donor sex							
Female n,%	102	43.0	39	54.2	63	38.2	0.02^a^
Donor ethnic group							
Asian n,%	19	8.0	6	8.3	13	7.9	NS
Black n,%	17	7.2	5	6.9	12	7.3	
Chin/oth/ns n,%	5	2.1	0	0.0	5	3.0	
White n,%	196	82.7	61	84.7	135	81.8	
Donor creatinine							
μMol/L, geo mean, SD	78.3	1.5	75.1	1.5	79.0	1.5	NS
Donor DM							
n,%	10	4.2	3	4.2	7	4.3	NS
Donor weight							
Kilograms, mean, SD	78.4	16.7	77.6	16.4	78.7	16.8	NS
Donor type							
ECD, n,%	64	26.0	42	25.5	22	27.2	NS
DBD, n,%	17	6.9	11	6.7	56	7.4	
DCD, n,%	71	28.9	20	24.7	51	30.9	
Live, n,%	94	38.2	33	40.7	61	37.0	
Mismatch level							
1, n,%	19	8.0	4	5.6	15	9.1	NS
2, n,%	52	21.9	14	19.4	38	23.0	
3, n,%	137	57.8	44	61.1	93	56.4	
4, n,%	29	12.2	10	13.9	19	11.5	
CIT (deceased donor only)							
Hours, mean, SD	16.1	4.5	16.4	4.1	16.0	4.6	NS
Indicated biopsies							
n, median, IQR	1	0–2	1.5	1–2.5	0	0–2	0.004^b^
Rejection							
n,%	24	9.8	7	8.6	17	10.3	NS
BK virus							
n,%	3	1.3	1	1.4	2	1.2	NS
Recipient eGFR at day 56							
mL/min/1.73 m^2^ median (IQR)	47.3	35.6- 59.7	50.1	35.7–65.4	46.4	33.8–57.6	NS

Rejection and indicative biopsies were counted up to the time of first post-transplant protocol biopsy in the group with protocol biopsies and to 12 wks in the group not undergoing a biopsy at this time point. Percentages represent column percentage. Numbers of deceased donors with CIT data: total 129; 38 and 91 in each group, respectively.

CIT, cold ischemic time; DM, diabetes mellitus; dysplastic, dysplastic kidneys; vasc/isch/hyp, renal disease classified as renovascular or due to chronic renal ischemia; ECD, extended criteria donor; DBD, donation after brain death (excluding ECD); DCD, donor after cardiac death; DBD, donor after brain death; SD, standard deviation.

a By chi-squared.

b By Wilcoxon rank-sum test.

### Predictors of ICD on implantation biopsy

Overall (including allografts where delta ICD was not available), median ICD0 was 2% (IQR 0–7). On univariable analysis, donor age, transplant type, and donor ethnic group were associated with tertile of ICD0 (Table 2). On multinomial logistic regression, there was a higher relative risk ratio of ICD0 falling in tertile two and three with both higher donor age and donation following cardiac death (Table 3). However other donor variables, including donor ethnic group, were not associated with tertile of ICD0 in the adjusted model. Furthermore, other than age, inclusion of factors that would lead to a donor being defined as “extended criteria” (ICH, serum creatinine >133 μMol/L, hypertension) did not alter the relative risk ratios. Most (six of nine) of the donors with DM were in the highest ICD0 tertile, but as overall numbers were small, this variable was not included in the multivariable model.

**Table 2:** Clinical variables stratified by ICD tertile at implantation

	ICD on implantation biopsy						p-Value
	Zero n = 72		Moderate n = 68		High n = 78		
ICD							
%, median, range	0	0–0	2	1–4	9	5–46	
Donor age							
Years, mean, SD	41.3	14.6	46.3	14.5	57.6	11.1	<0.001^c^
Donor sex							
Female n,%	35	48.6	28	41.2	28	36.8	NS
Donor ethnic group							
Asian n,%	8	11.1	9	13.2	1	1.3	0.02^d^
Black n,%	6	8.3	3	4.4	5	6.6	
Chin/oth/ns n,%	2	2.8	3	4.4	0	0.0	
White n,%	56	77.8	53	77.9	70	92.1	
Donor creatinine							
μMol/L, geo mean, SD	75.9	1.4	77.2	1.5	79.6	1.4	NS
Donor DM							
n,%	2	2.8	1	1.5	6	8.1	NS
Donor weight							
Kilograms, mean, SD	77.7	19.5	78.1	14.2	81.5	13.0	NS
Donor ICH							
n,%	22	30.6	17	25.0	26	33.3	NS
Donor type							
DBD n,%	29	40.3	15	22.1	27	34.6	0.001^a^
DCD n,%	11	15.3	21	30.9	33	42.3	
Live n,%	32	44.4	32	47.1	18	23.1	
CIT (DCD and DBD only)							
Hours, mean, SD	16.1	4.7	16.2	5.5	16.1	4.4	NS

Numbers of deceased donors with CIT data: total 115; 35, 32, and 48 in each group, respectively. Abbreviations and characters as Table 1.

CIT, cold ischemic time; DBD, donated after brain death; DCD, donation after cardiac death; DM, diabetes mellitus; ICD, index of chronic damage; ICH, intracerebral hemorrhage

c By analysis of variance (ANOVA).

d By Fisher’s exact test.

**Table 3:** Multinomial logistic regression model of ICD at baseline

	Risk ratio relative to lowest tertile ICD0					
	Middle			Highest		
	RRR	95% CI		RRR	95% CI	
Donor type						
DBD	Reference			Reference		
DCD	3.71	1.40	9.82	3.96	1.49	10.51
Live	1.93	0.87	4.33	0.77	0.31	4.32
Donor age						
Per decade >48 yrs	1.29	1.01	1.64	2.47	1.82	3.36

Generalized logistic regression model used as proportional odds assumption violated for the above variables across tertiles of ICD0.

ICD0, ICD at implantation biopsy; RRR, relative risk ratio; CI, confidence interval; DBD, donation after brain death; DCD, donation after cardiac death; ICD, index of chronic damage.

### Predictors of Change in ICD

**Figure 1:**
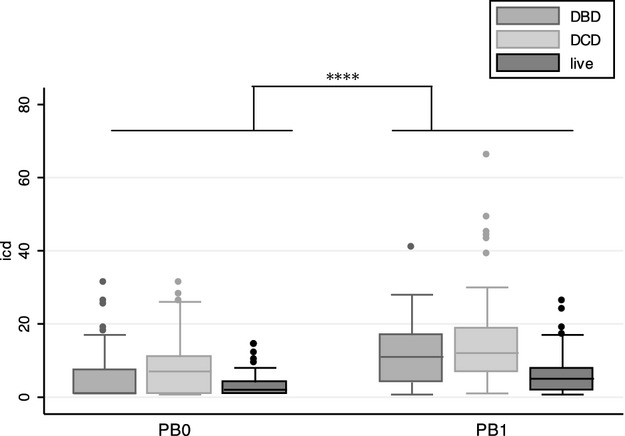
Index of chronic damage (ICD) at implantation and first protocol biopsy. Box and whisker plot of ICD scores at implantation (PB0) and ‘6–12 wk’ biopsy (PB1) stratified by donor type. Centre bar: median; Boxes: 25th and 75th centiles; Whiskers: upper and lower adjacent values; Dots: outliers. ****p < 0.001 by Wilcoxon signed-rank test. Includes allografts only where serial ICD was available.

Overall (including allografts where delta ICD was not available, n = 188), median ICD1 was 8% (IQR: 4–15). Restricting the analysis to those subjects for whom delta ICD was available, there was a small but highly significant increase between biopsies (median increase, 4%; IQR: 1–9). Box and whisker plots of ICD0 and ICD1 stratified by donor type are shown in Fig. 1. A negative delta ICD was observed in 25 of the 162 allografts.

**Figure 2:**
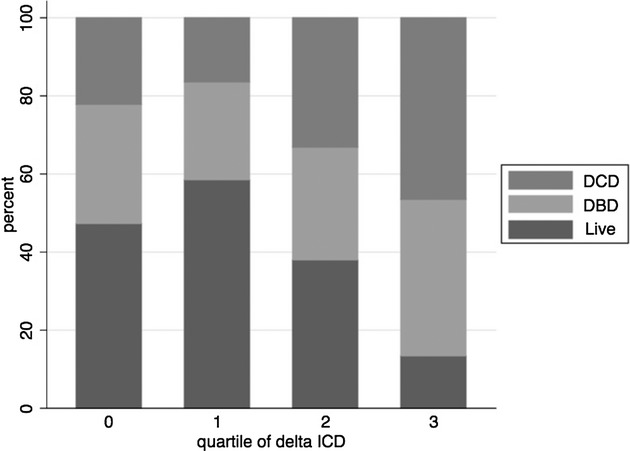
Prevalence of donor types amongst quartiles of delta index of chronic damage (ICD). Unadjusted for other clinical variables. ***p < 0.005.

Univariable associations between clinical variables and quartile of delta ICD are presented in Table 4. Transplant type (Fig. 2) and history of donor ICH along with recipient and donor age were the only clinical variables associated with delta ICD. Kidneys transplanted from live donors showed little change in ICD over time, while kidneys from DCD donors were more prevalent in the higher quartiles of delta ICD. Mean donor age was higher across increasing quartiles of delta ICD (p < 0.01 for a trend). Borderline relationships were found with donor serum creatinine concentration, donor ethnic group, previous transplantation, donor DM, and mismatch level, although the power to detect associations for the latter explanatory variables was limited due to small numbers.

**Table 4:** Clinical variables stratified by quartile of delta ICD on serial protocol biopsies

	Quartile of delta ICD								p-Value
	1 (n = 36)		2 (n = 36)		3 (n = 45)		4 (n = 45)		
Delta ICD									
%, Median, range	−2	−11 to 0	2	1 to 3	5	4 to 8	13	9 to 44	
Recipient age									
Years, mean, SD	52.4	11.5	47	14.5	47.2	13.4	54.1	12.1	0.023^c^
Recipient sex									
Female n,%	19	52.8	11	30.6	21	46.7	22	48.9	NS
Recipient ethnic group									
Asian n,%	5	13.9	6	16.7	11	24.4	8	17.8	NS
Black n,%	7	19.4	5	13.9	11	24.4	17	37.8	
Chin/oth/ns n,%	6	16.7	10	27.8	5	11.1	4	8.9	
White n,%	18	50.0	15	41.7	18	40.0	16	35.6	
Recipient primary disease									
Cystic n,%	2	5.6	3	8.3	3	6.7	4	8.9	NS
Dm n,%	3	8.3	4	11.1	5	11.1	3	6.7	
Dysplastic/reflux n,%	1	2.8	2	5.6	2	4.4	1	2.2	
Glomerular n,%	9	25.0	8	22.2	7	15.6	6	13.3	
Vasc/isch/hyp n,%	4	11.1	2	5.6	2	4.4	10	22.2	
Other n,%	14	38.9	10	27.8	14	31.1	12	26.7	
Unknown n,%	3	8.3	7	19.4	12	26.7	9	20.0	
Recipient DM									
n,%	6	16.7	5	13.9	7	15.6	6	13.3	NS
Previous transplant									
n,%	5	13.9	2	5.6	7	15.6	1	2.2	0.10^d^
Donor age									
Years, mean, SD	44.3	17.3	46.6	16.0	48.2	12.5	53.4	13.9	0.043^c^
Donor sex									
Female n,%	15	41.7	15	41.7	18	40.0	13	28.9	NS
Donor ethnic group									
Asian n,%	3	8.3	7	19.4	3	6.7	0	0.0	NS
Black n,%	3	8.3	3	8.3	2	4.4	4	8.9	
Chin/oth/ns n,%	1	2.8	1	2.8	1	2.2	2	4.4	
White n,%	29	80.6	25	69.4	39	86.7	39	86.7	
Donor creatinine									
μMol/L, geo mean, SD	72.4	1.4	84.2	1.4	75.3	1.3	86.3	1.6	0.10^c^
Donor DM									
n,%	0	0	0	0	2	4.4	4	9.1	0.10^d^
Donor weight									
Kilograms, mean, SD	77.4	17.4	78.5	16.3	79.7	17.1	80.4	14.7	NS
Donor type									
ECD n,%	5	13.9	6	16.7	12	26.7	18	40.0	<0.001^d^
DBD n,%	6	22.2	3	8.3	1	2.2	0	0	
DCD n,%	8	22.2	6	16.7	15	33.3	21	46.7	
Live n,%	17	47.2	21	58.3	17	37.8	6	13.3	
Donor ICH									
n,%	5	13.9	6	16.7	16	35.5	19	42.2	0.009^a^
Mismatch level									
1 n,%	4	11.1	2	5.6	7	15.6	2	4.4	0.11^d^
2 n,%	8	22.2	10	27.8	10	22.2	9	20.0	
3 n,%	19	52.8	19	52.8	28	62.2	26	57.8	
4 n,%	5	13.9	5	13.9	0	0.0	8	17.8	
CIT (DCD and DBD only)									
Hours, mean, SD	16.1	5.3	15.8	4.5	15.5	3.8	16.0	5.1	NS
Rejection									
n,%	5	13.9	5	13.9	6	13.3	7	15.5	NS
BK virus									
n,%	0	0	1	2.9	0	0	1	2.2	NS
Recipient eGFR at day 56									
mL/min/1.73 m^2^ median (IQR)	51.9	30.7–61.2	50.9	43.7–60.7	46.8	40.5–57.0	37.8	28.0–50.0	<0.001^e^
Recipient eGFR at day 365									
mL/min/1.73 m^2^ median (IQR)	52.2	40.9–73.8	52.6	40.9–73.8	51.9	41.6–59.1	45.1	35.0–52.1	0.04^e^

Numbers of deceased donors with CIT data: total 88; 15, 14, 27, and 32 in each group, respectively. Rejection was defined by a histologically proven episode of rejection prior to the protocol biopsy. Number of allografts with one-yr creatinine: 127. Abbreviations and characters as Tables 1 and 2.

CIT, cold ischemic time; DBD, donation after brain death; DCD, donation after cardiac death; DM, diabetes mellitus; ECD, extended criteria donor; ICD, index of chronic damage; ICH, intracerebral hemorrhage.

e By Kruskal–Wallis test.

Donor age and donor type were the only variables independently associated with quartile of delta ICD on multivariable analysis using an ordinal logistic regression model (Table 5). Increasing donor age was independently associated with increased odds of a higher quartile delta ICD with an OR of 1.29 (95% CI: 1.06–1.57) for each decade over 48 yr. Receiving a kidney from a live donor was independently associated with lower odds of a higher delta ICD quartile with an OR of 0.40 (95% CI: 0.20–0.80) compared with the reference group of DBD. There was no significant increase in odds associated with receiving a kidney from a DCD vs. DBD (OR 1.30; 95% CI: 0.62–2.73). Exclusion of donors dying from ICH did attenuate the differences in OR between live and DBD donors, suggesting that this may be one factor responsible for the differences seen in delta ICD between donor types (Table 5). Although the prevalence of donor DM appeared higher in the higher quartiles of delta ICD, again low numbers mean that this variable was not included in the final model. Other donor-, recipient-, and transplant-associated variables did not improve model fit. In particular, no associations were seen between episodes of rejection or length of CIT (in deceased donors only) and delta ICD.

**Table 5:** Multivariable ordinal logistic regression model of delta ICD quartile on serial protocol biopsies

	All donors n = 162			Donors with COD ICH excluded n = 116		
	OR	95% CI		OR	95% CI	
Donor type						
DBD	Reference			Reference		
DCD	1.30	0.62	2.73	2.90	1.00	8.39
Live	0.40	0.20	0.80	0.78	0.30	1.95
Donor age						
Per decade >48 yrs	1.29	1.05	1.57	1.33	1.05	1.60

Adjusted for timing of second biopsy (days since transplant). Model meets proportional odds assumptions.

COD, cause of death, OR, odds ratio; CI, confidence interval; DBD, donation after brain death; DCD, donation after cardiac death; ICD, index of chronic damage; ICH, intracerebral hemorrhage.

### Associations between delta ICD and biochemical measures of kidney function

There was a strong association between delta ICD and eGFR at day 56 (Table 4). There was also an association between delta ICD and quartile of eGFR at day 365 on multivariate testing (adjusted for episodes of rejection, recipient age, ethnic group, sex, and diabetes) when biochemical kidney function at the time of second biopsy was not included in the model. To test whether early fibrosis would be a useful predictor of future allograft function, we examined whether this measure was associated with the change in eGFR between day 56 and day 365. We found no association between delta ICD and change in kidney function between first post-transplant protocol biopsy and one yr post-transplant (quartile of eGFR at day 365 adjusted for quartile of eGFR at day 56; p = 0.74). Addition of potential confounders to the model (rejection, recipient factors) did not alter the lack of association.

## Discussion

In this study, we describe the clinical associations of early changes in chronic damage on protocol biopsies performed as a part of a routine clinical care. Although several other studies have examined the prevalence of chronic damage in routine biopsy samples, to our knowledge, this is the first one to examine early change in ICD outside a clinical trial, in a cohort including transplants from donors after cardiac and brain death, as well as live donors.

As with all studies of this type, a major concern is whether patients undergoing protocol biopsies are representative of the cohort of transplant patients as a whole. Therefore, we compared clinical variables in the group undergoing both protocol biopsies with the group undergoing only one or neither. We could only detect differences in donor sex and number of indicated biopsies. The higher number of indicated biopsies in the group not undergoing protocol biopsies may reflect causation (i.e., patients do not undergo protocol biopsies after a recent indication biopsy) or reverse causation (i.e., clinicians are more likely to refer for biopsy where there has not been a recent protocol biopsy). Reassuringly rates of biopsy proven rejection were no different, suggesting that our analysis of change in ICD is not restricted to a selected group of patients in this context. The reasons underlying lower numbers of female donors in the protocol biopsy group are less clear, but it could be that there are differences in transplant course in recipients of kidneys from female donors 12. Overall, these findings suggest that the cohort in whom we examined delta ICD is broadly reflective of the transplant population followed up at our institution during the same time period.

Donor age was independently associated with ICD0 underlining the well-recognized importance of this variable in considering the suitability of organs for transplantation. Furthermore, DCD kidneys had a higher baseline ICD independent of age, which may reflect a difference in the underlying clinical characteristics of donors of kidneys of these types accepted at our institution. No differences in baseline ICD were observed between kidneys from live donors and DBD after adjustment for donor age. Others have reported associations between early changes in fibrosis and diabetes, rejection, and kidneys from male donors 9. We observed trends with the first two of these risk factors and delta ICD in our cohort, but may have been underpowered to detect significant associations. Furthermore, kidneys from female donors made up less than 30% of those allografts in the highest delta ICD quartile compared with more than 40% in the lowest three quartiles; however, this association did not reach statistical significance using our approach.

Younger donor age and live donation were independently associated with reduced odds of higher delta ICD. As age is known risk factor for failure to recover from acute kidney injury 13, it might be expected to impact on recovery from transplantation-associated ischemia–reperfusion injury. In one similar study, a similar association was found on univariable testing 9. Another recent study did not observe a similar association with donor age 8; this may be because this investigation was restricted to live donors, where the increase in ICD is not marked, limiting the power to detect any relationship. Although live donors are often younger than DBD or DCD, the association of live donation with lower delta ICD was independent of donor age.

Overall, there was a small, but highly significant increase in ICD between biopsies. This increase in chronic damage in our cohort appears consistent with what has been reported in other studies [8, 9]. This early increase in chronic damage suggests that the process of transplantation itself leads not only to an acute kidney injury, but also to irreversible damage detectable at 2–3 months.

Despite differences in ICD at implantation, there was no evidence for a difference in delta ICD between DBD and DCD after adjustment for donor age, suggesting that although kidneys from DCD in our cohort may have higher levels of chronic damage prior to transplantation, the post-transplant progression of this damage is similar between these two donor types. We also found no evidence that either CIT (within the range of CIT seen in deceased donors) or early acute rejection was associated with delta ICD. It is impossible to confidently separate the impact of CIT from donor type in a study of this design, but these data suggest that there may be non-ischemic mediators of the association between live donors and lower delta ICD. The attenuation of the differences between live and DBD donors after exclusion of donors dying from ICH may implicate this cause of donor death in aggravated transplantation-associated allograft injury and is consistent with the worse medium-term outcomes observed in kidneys transplanted from such donors 14. Our cohort included too few donors with DM to draw adequate conclusions as to any association between this risk factor and delta ICD; however, with the increasing use of diabetic donors, this aspect of donor selection would merit further investigation.

We examined associations between delta ICD (as the explanatory variable) and eGFR, both at the time of second biopsy and at one yr. Unsurprisingly, delta ICD was associated with eGFR at both time points. These finding are similar to those reported by Servais and colleagues 9. However, change in ICD between implantation and first post-transplantation biopsy was not additionally informative of biochemical outcome at one yr (a surrogate for graft loss 2) over and above an eGFR measured at the time of the latter biopsy. This analysis does have potential for confounding as clinicians were aware of ICD scores and may have altered patient management accordingly, and the small numbers of transplants lost in the period between first post-transplant biopsy and end of follow-up mean that we are unable to examine hard outcomes such as graft failure. However, these findings do suggest that although early change in chronic damage reflects past insults, this measure is unlikely to provide useful information (above routine biochemical measures) on the future transplant course as judged by change from eight-wk to one-yr eGFR.

Almost 16% of serial protocol biopsies demonstrated a negative delta ICD. Areas of chronic damage are not thought to recover, so these values are unlikely to represent true changes. A similar finding has been reported elsewhere 15, and as interobserver variability and intra-observer variability in the reporting of ICD have been found to be low 7, negative scores are likely to reflect sampling variation. This implies that although potentially a useful research tool, for an individual, even serial ICD measurements are likely to be of limited utility except perhaps for very high values 7.

In addition to those mentioned above, our study has several other weaknesses. Although we examined, and found little evidence for, differences between the group undergoing protocol biopsies and those who did not, we cannot exclude the possibility that we have examined a selected group of patients. All our patients were from a single center, and therefore, our conclusions may not be generalizable. Furthermore, we did not examine our biopsies for inflammation in scarred areas, a finding that has been reported to be a useful predictor of adverse graft outcomes 4. Although, to our knowledge, this is the largest study examining changes in ICD in recipients of kidneys from unselected donors, it is possible that it was underpowered to detect all the important relationships of delta ICD. However, with more than 50 transplants of each donor type, we would expect to identify most clinically relevant associations.

In summary, this study demonstrated an early, small, but measurable increase in irreversible damage in allograft kidneys over the first three months following transplantation. This increase in chronic damage was associated with donor age. Furthermore, transplantation from a live donor was associated with a smaller increase in ICD. The routine clinical value of measurement of chronic damage is unclear due to both the potential for sampling variation and the observation that the early increase in histological damage appears to reflect past insults, but not future course as judged by biochemical change in function over the following nine months.
